# Valorisation of tomato pomace in anti-pollution and microbiome-balance face cream

**DOI:** 10.1038/s41598-024-71323-4

**Published:** 2024-09-03

**Authors:** Katarzyna Rajkowska, Anna Otlewska, Aleksandra Raczyk, Ewa Maciejczyk, Agnieszka Krajewska

**Affiliations:** 1 Faculty of Biotechnology and Food Sciences, Institute of Fermentation Technology and Microbiology, Lodz University of Technology, Wólczańska 171/173, 90-530 Łódź, Poland; 2 Faculty of Biotechnology and Food Sciences, Institute of Natural Products and Cosmetics, Lodz University of Technology, Wólczańska 171/173, 90-530 Łódź, Poland

**Keywords:** Tomato pomace oil, Face cream, Facial skin microbiome, Antipollution effect, Fatty acid profile, Volatile compounds, Biotechnology, Microbiology

## Abstract

Tomato pomace, the main by-product of tomato processing, is also an underestimated source of many active substances. This study aimed to determine the possibility of using oil obtained from tomato pomace in a face cream formulation. The bacterial community structure, face skin biophysical parameters and protection against air pollution were examined after daily application of the cosmetic by volunteers. In the tomato pomace oil, the profile of fatty acids was determined by GC‒MS, and the profile of volatile compounds was determined using the HS-SPME technique. The dominant bioactive component in the oil was linoleic acid (63.6%), and among the volatile compounds, it was carvotanacetone (25.8%). The application of the cream with tomato pomace oil resulted in an increase in the dominant genera *Staphylococcus*, *Anaerococcus* and *Cutibacterium* in the epibiome, particularly beneficial *Staphylococcus epidermidis,* while limiting the growth of the potentially opportunistic pathogens *Kocuria* spp., *Micrococcus* spp., *Veillonella* spp., and *Rothia* spp. This study showed the usefulness of tomato pomace oil as a natural ingredient in skin care cosmetics, reducing skin inflammation, sensitivity and melanin level, with potential protective effects against air pollution and microbiome-balance properties. Tomato pomace, which is commonly considered waste after tomato processing, can be used in the development of new cosmetics and may additionally contribute to reducing environmental nuisance.

## Introduction

On a global scale, the production of fresh tomatoes amounted to 186 million tonnes (Mt) in 2022 and is constantly increasing^1^. Globally, an average of 39 Mt of tomatoes are cultivated for the processing industry, with the world's leading processing countries being California (9.5 Mt), China (6.2 Mt), Italy (5.5 Mt), Turkey (2.4 Mt), and Spain (2.1 Mt)^2^. The main by-product after processing tomatoes is tomato pomace, which is composed of peels (almost 50%), seeds (approximately 40%) and a small part of the pulp^3^. These residues usually account for 2–3% to as much as 5% of the processed raw material^4^, which means the production of up to 2 Mt of tomato pomace per year.

Currently, tomato pomace is mainly used as animal feed; in the production of compost, it is dumped in landfills or even burned, contributing to carbon dioxide emissions and the burden on the environment^5^. On the other hand, several studies have shown the rich composition of tomato processing by-products such as carotenoids, phenolic compounds, ascorbic acid, dietary fibre, proteins, and polyunsaturated fatty acids, which can be beneficial to both human nutrition and health^3,4,6,7^. Moreover, it was reported that the fractions of seeds and peels contributed the most to the antioxidant activity of tomatoes^8^. It seems that tomato pomace is an underestimated source of nutrients and bioactive compounds, so its use can be both a response to the growing consumer demand for health-beneficial ingredients and valuable from an environmental perspective.

According to WHO data, almost all of the global population (99%) is exposed to air pollution exceeding the limits of the WHO guidelines^9^. Exposure to high levels of air pollutants can cause a variety of health outcomes, affecting a number of different systems and organs. It increases the risk of acute respiratory infections in children and chronic bronchitis in adults, asthma, chronic obstructive pulmonary disease, ischaemic heart disease, and lung cancer^10^. Airborne pollutants have also been associated with diseases of the central nervous system, including stroke, Alzheimer's disease, Parkinson's disease, and neurodevelopmental disorders^11^. Moreover, both short- and long-term exposure to air pollution have been associated with premature mortality and reduced life expectancy. The most harmful health pollutants are particles less than 2.5 µm in diameter (PM_2.5_) because they can be inhaled into and accumulate in the respiratory system (WHO, 2021). Nanosized particles can also translocate to the central nervous system and activate innate immune responses^11^.

One of the major targets of air pollutants is human skin, which is the largest organ of the human body and acts as a barrier. The main air pollutants affecting the skin include ultraviolet radiation, polycyclic aromatic hydrocarbons, volatile organic compounds, nitrogen oxides, particulate matter, and cigarette smoke, which exert various toxicological effects on the skin^12^. Air pollutants may interfere with the normal functions of lipids, DNA, and proteins in human skin through oxidative damage, leading to the degradation of collagen, skin aging, inflammatory or allergic skin diseases such as atopic dermatitis, psoriasis and acne, and skin cancer^12,13^.

Three pivotal strategies can be used to protect human skin against environmental pollution, i.e. first of all, proper skin cleansing as the basis of an effective anti-pollution routine, next creating a physical barrier on the skin surface through the use of film-forming ingredients in cosmetics. The third way is the inclusion of antioxidants in cosmetic recipes to protect the skin against free radicals^14^. In this aspect it can be assumed that tomato pomace, which is characterized by high antioxidant activity, has the potential to be used as a functional ingredient in antipollution skincare products.

This study was undertaken to verify the usefulness of tomato pomace oil as a key ingredient in face cream recipe in terms of its skincare and protective properties. In addition, since the proper functioning and barrier properties of the skin are closely related to the skin microbiota^15^, it is crucial to determine the effect of cosmetics on this microbiome, which was also the subject of our study.

## Results

### Effect of the cream with tomato pomace oil in a test with volunteers

**Fig. 1:**
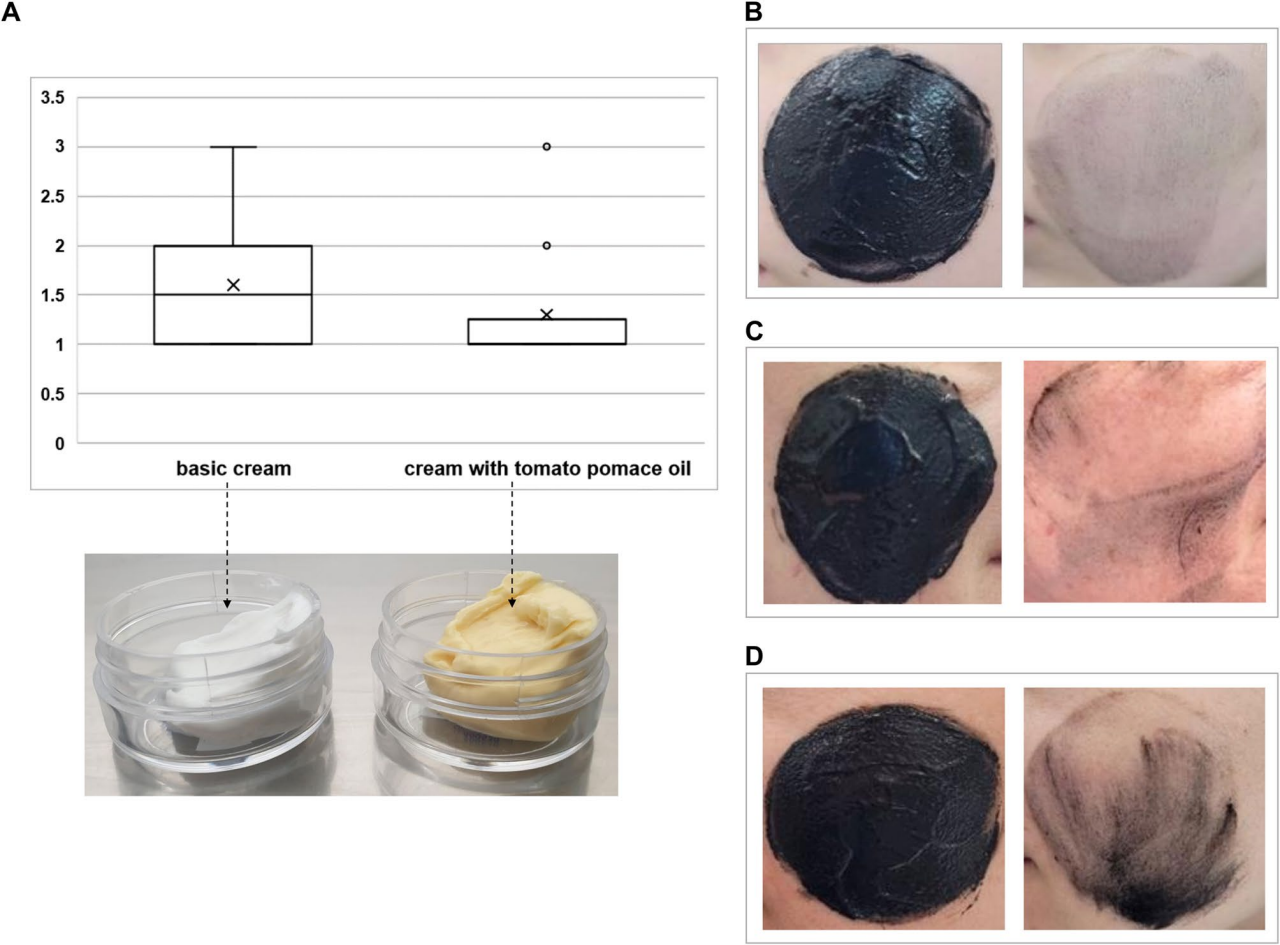
Limitations of particulate pollutant penetration on the face skin surface after a 7-day application of basic cream and cream with tomato pomace oil (**A**) and visual assessment (B-D) on a 3-point scale: 1—the skin surface remained slightly grey with single visible contaminated pores (**B**); 2—the skin surface remained slightly grey with more intense local residues of pollutants and visible contaminated pores (**C**); 3—the skin surface remained visibly grey with very intense local residual pollutants and visible contaminated pores (**D**).

In this study, a previously developed sebum pollution model imitating a very robust and exaggerated surrogate for dirty, polluted skin was used^16^. It was assumed that a cream with antipollution properties should protect against the deposition of particulate pollutants on the skin surface and penetration into the skin pores; therefore, the sebum pollution model should be easier to remove. Compared with the basic cream, the application of face cream with tomato pomace oil twice a day for 7 days reduced the penetration of pollutants (mean 1.3 versus 1.6) (Fig. 1). Since the face cream was intended to be universal and adaptable to different users, the group of volunteers participating in the study was also diverse in terms of gender and age. This resulted in very diverse skin parameters but also in individual reactions to the cream. For three out of ten participants, the addition of tomato pomace oil to the face cream provided significantly better protection against contaminants than did the basic cream (Fig. 1). The results indicate the usefulness of tomato pomace oil as an ingredient in cosmetic formula that may enhance its antipollution effect.

**Fig. 2:**
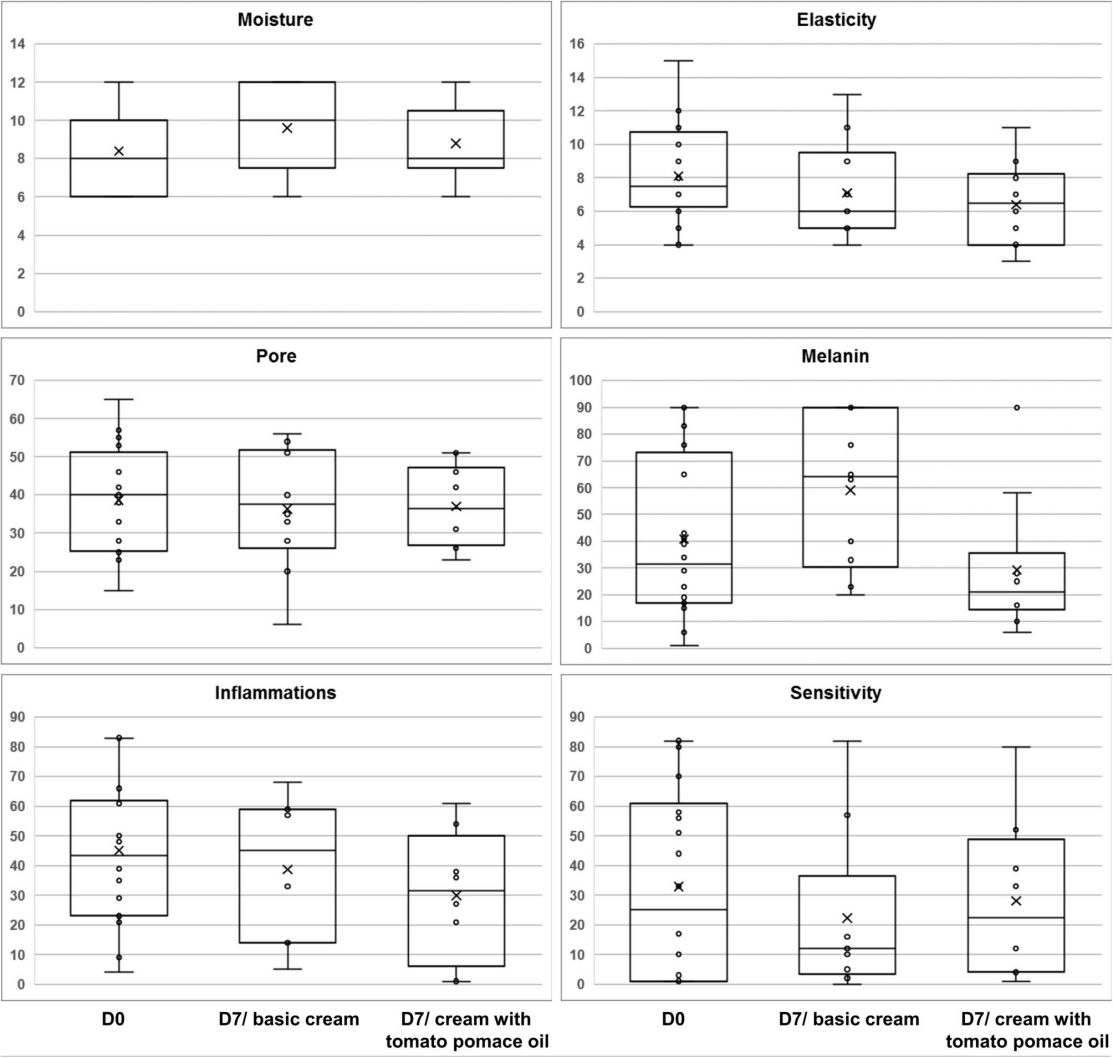
Effects of the basic cream and cream with tomato pomace oil on skin biophysical parameters based on the results obtained for 10 participants.

At the start and after 7 days of using the face creams, biophysical parameters, including facial skin moisture, elasticity, pore, melanin, inflammation, and sensitivity, were measured (Fig. 2). The skin hydration level increased after regular use of both the basic cream and the cream with tomato pomace oil by an average of 14.3 and 4.8%, respectively. Skin sensitivity and inflammation also improved during the test period. These parameters decreased on average by 32% and 14% following the use of basic cream and by 14% and 33%, respectively, following the use of cream with tomato pomace oil (Fig. 2). A reduction in skin sensitivity was noted for 5 participants using the basic cream and for 6 participants after application of the cream with tomato pomace oil.

For both tested cosmetics, the pore visibility remained almost unchanged throughout the evaluation period. However, the average melanin content increased significantly after the use of the basic cream, from 40.9 to 59.0. Such an effect was not noted for the cream with tomato pomace oil, the use of which resulted in unifying the skin tone (mean equal to 29.2). Moreover, the use of the tested creams adversely affected the elasticity of the participants' skin, reducing this parameter by an average of 12.3% (basic cream) and 20.9% (cream with tomato pomace oil). Notwithstanding, 5 participants showed an increase in skin elasticity after regular application of the basic cream, and 2 volunteers showed an increase after using the cream with tomato pomace oil.

The results indicated moisturizing, soothing, and skin tone-unifying properties of the tested face creams. These features partially resulted from the basic composition of the creams, but the addition of tomato pomace oil clearly reduced inflammation, skin sensitivity and tonality.

### Profile of fatty acids and volatile compounds in tomato pomace oil

Both the antipollution effect and the impact on the biophysical skin parameters of the cream with tomato pomace oil are related to the composition of the oil. The oil content of the tomato pomace was 7.11 ± 0.58%, and the fatty acid composition is presented in Table 1. The dominant fatty acid in the oil was linoleic acid, with an average content of 63.6%. The content of monounsaturated acids in the tomato oil was determined by the amount of oleic acid (14.9%), and the amount of saturated acid was dependent on the amount of palmitic (11.6%) and stearic acid (3.5%). The analysed sample also contained linolenic acid (2.1%), while arachidic acid (0.38%) and palmitoleic acid (0.32%) were present in smaller amounts. Margaric and myristic acids were also detected, but in trace amounts (0.15% and less than 0.1%, respectively).

**Table 1:** Concentrations of fatty acids in the tested tomato oils.

Fatty acid		Content (mg/g oil)	Relative concentration (%)
C14:0	Myristic acid	0.02 ± 0.002	0.09 ± 0.01
C16:0	Palmitic acid	1.89 ± 0.022	11.57 ± 0.13
C16:1	Palmitoleic acid	0.05 ± 0.007	0.32 ± 0.04
C17:0	Margaric acid	0.03 ± 0.001	0.15 ± 0.01
C18:0	Stearic acid	0.57 ± 0.008	3.48 ± 0.05
C18:1	Oleic acid	2.44 ± 0.013	14.90 ± 0.08
C18:2	Linoleic acid	10.40 ± 0.021	63.57 ± 0.12
C18:3	Linolenic acid	0.34 ± 0.052	2.06 ± 0.30
C20:0	Arachidic acid	0.06 ± 0.003	0.38 ± 0.02

During GC-FID-MS analysis of volatile compounds in tomato pomace oil, 48 different constituents were identified, accounting for 84.7% of all volatiles (Table 2). Terpenoids, phenylpropanoids and nonterpene compounds were found in the analysed sample. The percentage of compounds from the last group was the largest (41.6%). Among the nonterpenoids, ketones (e.g., 6-methyl-5-hepten-2-one, 9.2%; heptan-3-one, 4.6%; 6-methyl-3,5-heptadiene-2-one, 2.5%; undecane-2-one, 0.5%); alcohols (6-methylhept-5-en-2-ol, 7.7%; β-phenylethanol, 3.3%); aldehydes (e.g., benzaldehyde, 1.4%; nonanal, 1.4%; non-2-enal, 1.4%); and acids (e.g., hexanoic acid, 3.6%; heptanoic acid, 1.5%) were present.

**Table 2:** Composition of volatile compounds in tomato pomace oil analysed by the HS-SPME-GC‒MS technique.

Compound	RI lit	RI exp	Content (%)	Compound	RI lit	RI exp	Content (%)
Heptan-3-one	887	886	4.6	Carvotanacetone	1218	1217	25.8
α-Pinene	934	933	t	Chavicol	1219	1223	0.1
Benzaldehyde	941	933	1.4	Piperitone	1226	1228	0.3
6-Methyl-5-hepten-2-one	968	964	9.2	3-Methylpentyl angelate	1230	1237	0.4
Dehydro-1,8-cineol	973	970	0.7	Linalyl acetate	1239	1241	0.8
6-Methylhept-5-en-2-ol	981	978	7.7	Geranial	1243	1244	0.4
Hexanoic acid	996	993	3.6	Nonanoic acid	1260	1257	0.2
Heptanoic acid	1074	1070	1.5	Bornyl acetate	1270	1270	3.2
6-Methyl-3,5-heptadiene-2-one	1088	1079	2.5	Undecan-2-one	1273	1275	0.5
Nonanal	1084	1084	1.4	Menthyl acetate	1280	1278	0.3
β-Phenylethanol	1085	1086	3.3	α-Methylnaphtalene	1298	1290	0.1
Hexanamide		1117	1.8	Tridecane	1300	1301	0.3
Menthone	1136	1134	1.0	Bicycloelemene	1333	1333	0.8
Non-2-enal	1145	1136	1.4	Citronellyl acetate	1338		t
Isomenthone	1146	1143	0.8	Butyl caprylate		1356	0.6
Borneol	1150	1152	0.3	Methyleugenol	1369	1372	0.2
Cryptone	1160	1157	0.3	α-Copaene	1380	1377	1.0
Neomenthol	1156	1159	2.3	Cyperene	1402	1401	0.2
p-Cymen-8-ol	1169		t	β-Caryophyllene	1421	1419	0.3
Terpinen-4-ol	1164	1163	1.8	Geranyl acetone	1430	1430	0.8
α-Terpineol	1176	1174	0.4	α-Ionon-5,6-epoxide	1467	1462	0.1
Methylchavicol	1176	1177	0.9	β-Ionone	1468	1465	0.3
Decanal	1180	1186	0.5	ar-Curcumene	1473	1472	0.2
Cumin aldehyde	1215	1212	0.2	Actinidiolide	1495	1489	1.1
Total identified							84.7
Monoterpene hydrocarbons							< 0.05
Oxygenated monoterpenes							39.3
Phenylpropanoids							1.4
Sesquiterpene hydrocarbons							2.5
Nonterpenoids							41.6

*RI lit* retention index according to the literature, *RI exp* experimental retention index, *t* trace < 0.05%.

In addition to this compound, other oxygenated monoterpenes, such as bornyl acetate (3.2%), neomenthol (2.3%), menthone (1.0%), terpinene-4-ol (1.8%), and isomenthone (0.8%), were also detected. Sesquiterpene and monoterpene hydrocarbons were also detected in the headspace of tomato pomace oil. The content of sesquiterpene hydrocarbons was only a few percent. Among this group, the following compounds have been identified: bicycloelemene (0.8%), α-copaene (1.0%), cyperene (0.2%), β-caryophyllene (0.3%) and ar-curcumene (0.2%). The content of monoterpene hydrocarbons was also very low. There was only one compound detected among this group (α-pinene < 0.05%). No oxygenated derivatives of sesquiterpenes were identified in the analysed sample. Interestingly, the volatiles in tomato pomace oil were phenylpropanoids (1.4%), represented by methyleugenol (0.2%), chavicol (0.1%), methylchavicol (0.9%), and cumin aldehyde (0.2%).

### Epibiome structure of facial skin

**Fig. 3:**
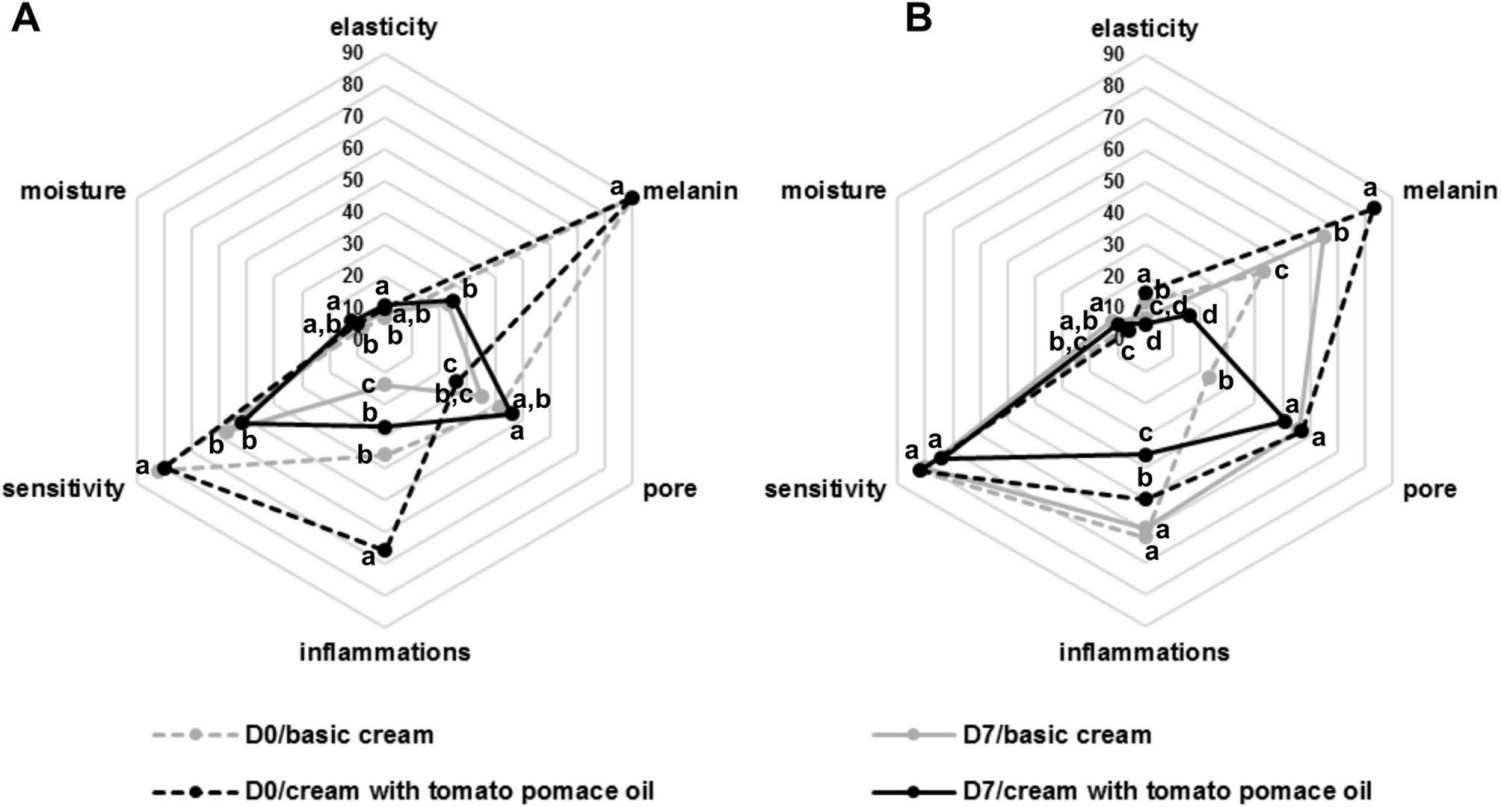
Changes in the skin biophysical parameters of one female (**A**) and one male participant (**B**) selected for further studies of the facial skin microbiota structure. Means within individual parameters followed by different letters are significantly different.

The bacterial community was analysed for two participants, who showed substantial changes in skin parameters, as well as strong antipollution effects after applying the cream with tomato pomace oil (Fig. 3). After a 7-day application of both creams, the skin elasticity of the selected female participants increased by 29% after the application of the basic cream and by 10% after the application of the cream with tomato pomace oil; moreover, the sensitivity decreased by 30% and 35%, respectively, and the moisture content increased by 25% and 20%, respectively. Both creams also helped to reduce inflammation by approximately 60% and melanin levels by more than 70% (Fig. 3A). For the selected male participant, marked changes in moisture (increases of 50% and 67%, respectively), elasticity (decreases of 42% and 67%, respectively) and melanin levels (increases of 51% after the use of basic cream but decreases of as much as 81% after the use of cream with tomato pomace oil) were observed. Both creams also showed a beneficial effect on reducing inflammation and the sensitivity of the facial skin (Fig. 3B). Unlike most volunteers, these participants showed a significant increase in pore size after using basic cream (male) or cream with tomato pomace oil (female).

We aimed to estimate the differences in the microbiome composition of facial skin using high-throughput sequencing before (D0M—male, D0F—female) and after treatment with the basic cream (D7M_BC and D7F_BC) and the cosmetic with tomato pomace oil (D7M_TC and D0F_TC). A total of 443,742 raw reads were obtained for both samples, and the number of classified reads was greater (171,520) for the T0F sample than for the T0M sample (155,552). The degree of microbial diversity expressed as the Shannon index, calculated for bacterial genera, was similar for the female and male skin microbiomes and reached 2.3 and 2.4, respectively.

**Fig. 4:**
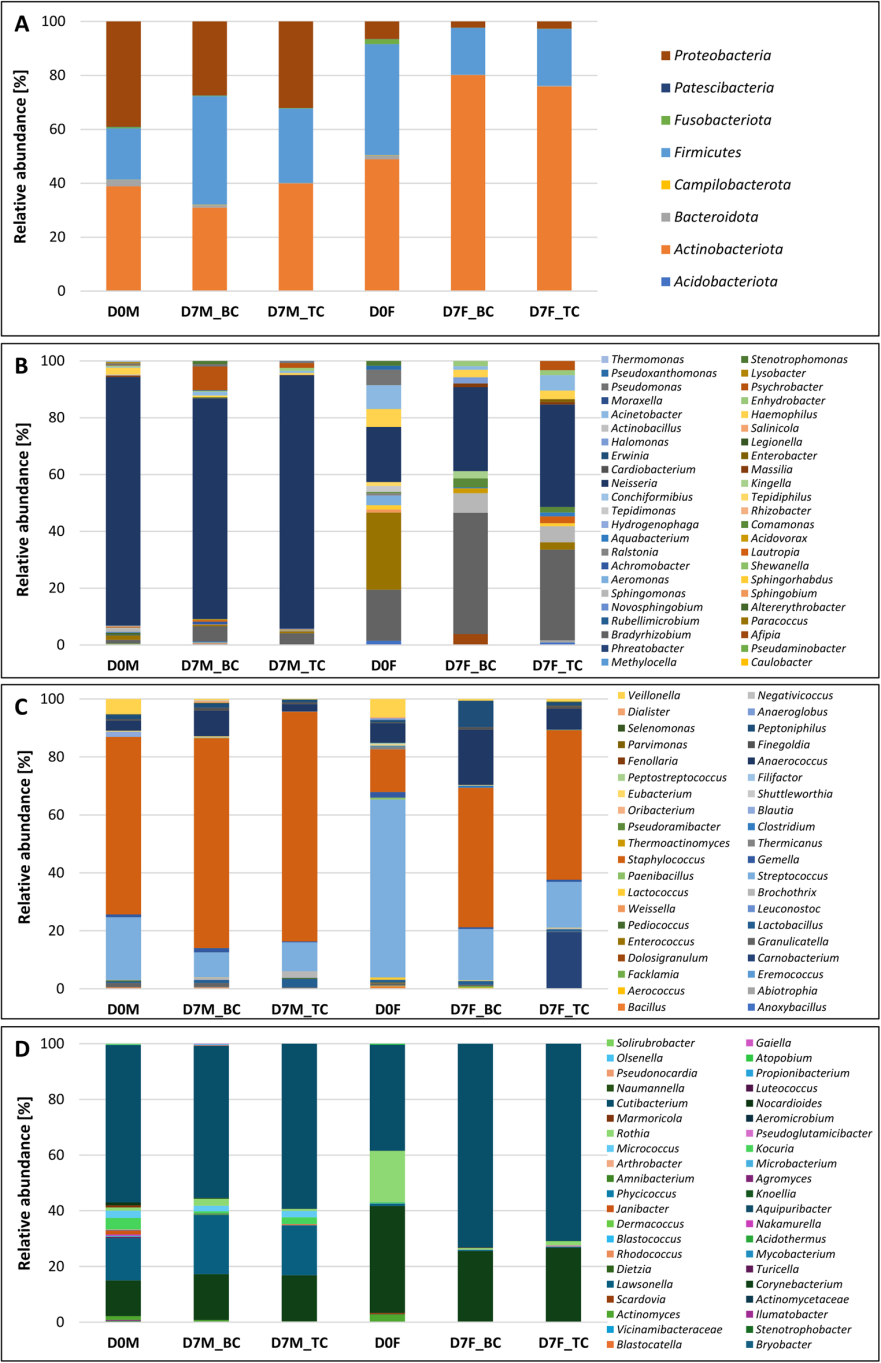
Comparison of the epibiome structure following the use of face creams. Relative abundance of bacteria at the order level (**A**) and genus level in *Proteobacteria* (**B**), *Firmicutes* (**C**), and *Actinobacteriota* (**D**).

According to the classification data, 93 bacterial genera were detected in the microbial community of male facial skin before cream treatment, while 69 genera were detected in female facial skin. The skin microbiome was composed of 8 phyla: *Acidobacteriota, Actinobacteriota, Bacteroidota, Campilobacterota, Campilobacterota, Firmicutes, Fusobacteriota, Patescibacteria*, and *Proteobacteria* (Fig. 4A). *Firmicutes, Actinobacteriota* and *Proteobacteria* were found to be the most abundant in the facial skin microbiome, with differences in the percentages of each phylum depending on the volunteer and a significant change after the application of basic and tomato pomace oil creams (Fig. 4B–D).

*Actinobacteriota* and *Proteobacteria* were present in very similar proportions (38.6% and 38.9%, respectively) and were the most abundant phyla in the male facial skin samples before creams application. In the female skin microbiome, *Actinobacteriota was* also the predominant group (48.8%), followed by *Firmicutes* (41.2%) and *Proteobacteria,* which accounted for only 6.5% of the total OTUs (Fig. 4B–D).

Among the *Actinobacteriota, Cutibacterium* spp. (*C. acnes, C. avidum*) was dominant in all the samples and accounted for 18.8–21.3% (Figs. 4D, 6A,B). A similar percentage (18.8%) was noted for *Corynebacterium* (mainly *C. kroppenstedtii*) in the case of the female skin microbiome, while in the male skin microbiome, its frequency was less than 5%. In the same sample (D0M), *Lawsonella* spp. were detected at a similar level (5.7%). In turn, their presence in the female skin microbiome did not exceed 0.5%.

The *Firmicutes* phylum was represented mainly by bacteria belonging to three genera, namely, *Streptococcus* (*S. anginosus, S. gordonii, S. mutans, and S. salivarius*) and *Staphylococcus*, followed by *Anaerococcus*. In the D0F sample, the first of the aforementioned was classified as 25.8%, while in the D0M sample, it was calculated to be 4.1%. In contrast, *Staphylococcus* spp. (*S. aureus, S. epidermidis, S. equorum, S. lugdunensis, and S. vitulinus*) were the predominant genera (11.5%) in the male skin microbiome, while they constituted 6.2% of the female skin microbiome. In both samples, gram-positive bacteria belonging to *Anaerococcus* spp. (*A. hydrogenalis, A. prevotii*) in the range 0.7–2.9% were detected. Other bacterial genera did not exceed 2% in the whole facial skin microbiome profiles (Figs. 4C, 6A,B).

Overall, the individual bacterial genera affiliated with *Proteobacteria* (mainly *Gammaproteobacteria*) accounted for less than 3%. Among them, bacteria commonly occurring on human skin, such as *Haemophilus* (0.27–0.93%), *Acinetobacter* (0.1–0.35%), *Enhydrobacter* (0.1–0.15%), *Moraxella* (0.3–0.4%) and *Pseudomonas* (0.2%), were found. The exception was the genus *Neisseria*, which constituted 33.3% of the whole microbiome in sample D0M (Figs. 4C, 6A,B).

### Effect of the cream with tomato pomace oil on the facial skin microbiome

**Fig. 5:**
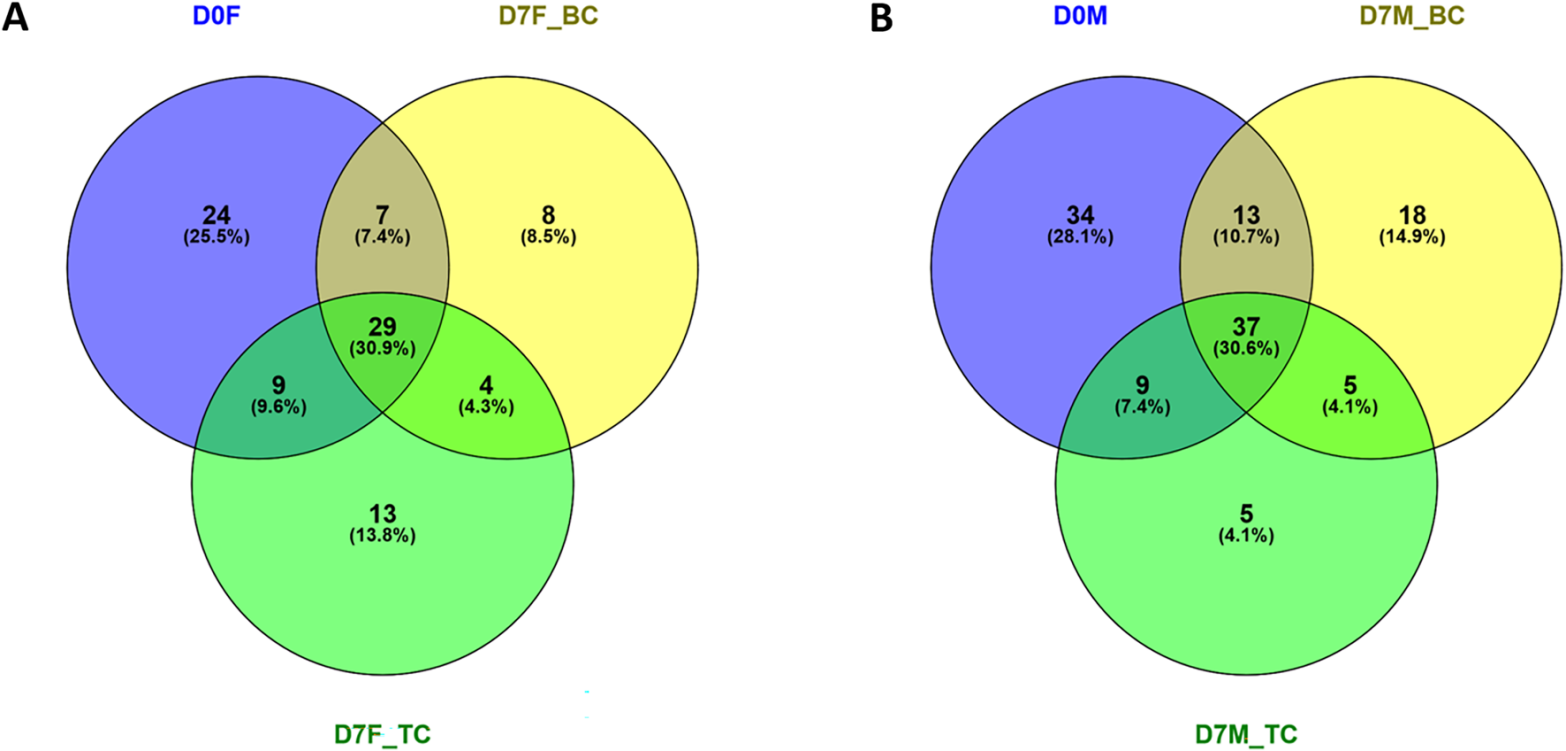
Venn diagrams representing changes in genus number after face creams application in female (**A**) and male (**B**) volunteers.

To assess the differences in the facial skin microbiome after creams application, the structure of the microbial community was compared, and the Shannon biodiversity index was determined. The number of bacterial genera included in the male and female skin microbiomes after treatment with the basic product decreased to 73 and 48, respectively (Fig. 5A,B). Interestingly, after using the cream with tomato pomace oil, the number of genera was the same in both specimens, amounting to 56, and decreased compared to that in the samples before cream application. The Shannon index indicated a significant reduction in the biodiversity of the skin microbiome after using the cream with tomato pomace oil, both in relation to the state before the creams application and after applying the basic product. For sample D7F_PC from the female skin bacterial community, this value was 1.6, while for the male skin bacterial community (D7M_PC), it was 1.4.

Both the basic cream and the cream with tomato pomace oil caused changes in the percentages of the three main phyla. In the case of male facial skin, after using the basic product, an increase in *Firmicutes* and a simultaneous reduction in *Actinobacteriota* and *Proteobacteria* were detected. In contrast, in the female skin microbiota, a significant increase was observed only for *Actinobacteriota*, while the number of *Firmicutes* and *Proteobacteria* decreased twofold for both the base and enriched creams (Fig. 4A).

**Fig. 6:**
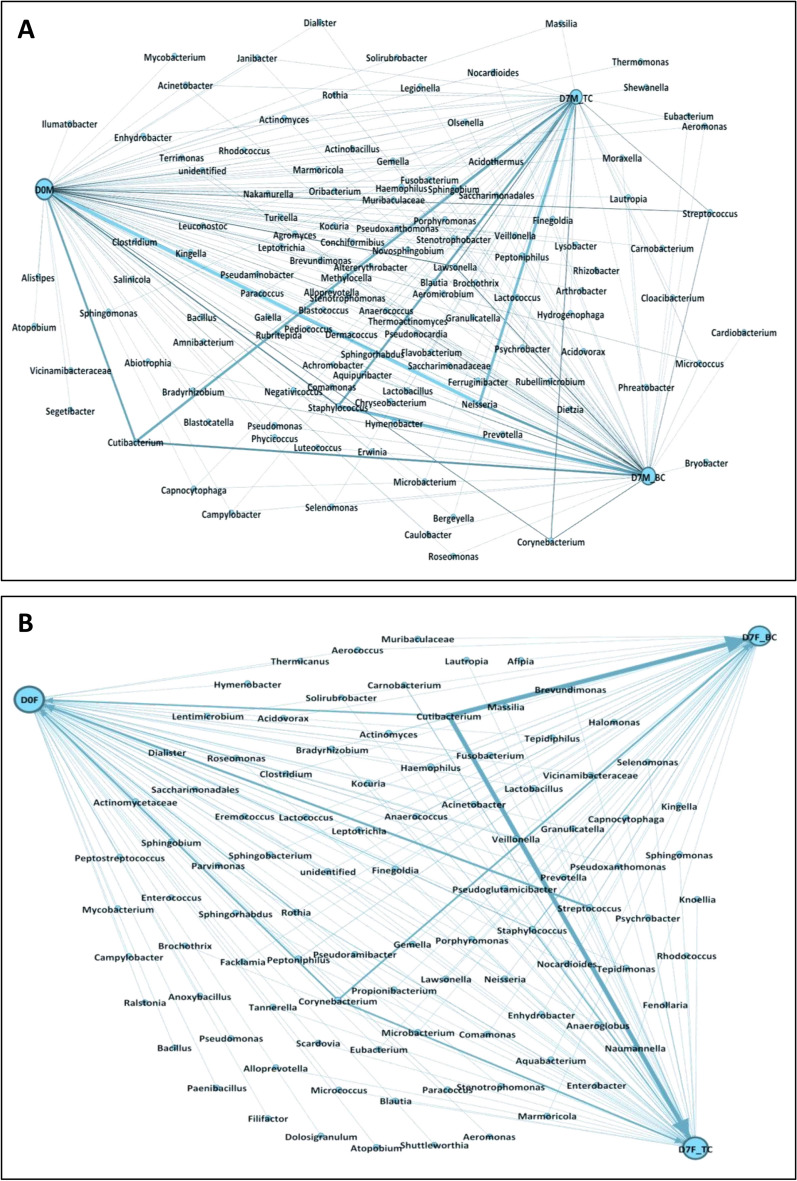
Bacterial co-occurrence network in female (**A**) and male (**B**) epibiomes after creams application.

After applying each of the creams, the profile of the dominant species of the facial microbiome also changed. The percentage of *Streptococcus* (*S. gordonii, S. salivarius, and S. mutans*) decreased more than threefold for male facial skin (D7M_PC) and fourfold for female facial skin (D7F_PC). A similar phenomenon was observed for *Veilonnella* spp., whose number decreased significantly regardless of the type of applied cream. In contrast, both samples showed increases in the abundance of *Staphylococcus* spp. (mainly *S. epidermidis*) of over 18% and 35%, respectively (Figs. 4C, 6A,B). An interesting relationship was revealed for *Anaerococcus* bacteria. The relative abundance of these genera increased from 3 to 8% for males and from 6 to 19% for females after treatment with the basic cream, while after treatment with the cream containing tomato pomace oil, the relative abundance of these genera remained at the same level as that in the initial state (Fig. 6A,B). The use of both creams changed the abundance of *Corynebacterium* spp.*, Lawsonella* spp.*,* and *Cutibacterium* spp. The abundance of *Corynebacterium* and *Lawsonella* increased independently of the cream type in the samples collected from the man (D7M_BC and D7M_PC). In contrast, in the case of the woman, the percentage of the aforementioned bacteria decreased. In turn, the level of *Cutibacterium* increased in female facial skin samples, while in male skin samples, it remained at a similar level (Fig. 6A,B). A significant change in the number of OTUs was also detected for the bacterial genera *Kocuria, Micrococcus,* and especially *Rothia* after the tomato pomace cream treatment. The percentage of *Rothia* spp. in the female facial skin sample decreased from 18.3% (D0F) to 1.5% (D7F_PC). Despite the changing relative abundance of individual bacterial groups, none of important species such as *Cutibacterium*, *Corynebacterium* and *Staphylococcus* was completely eliminated after the application of the cream with tomato pomace oil (Fig. 5A,B). This is crucial in terms of interactions between microorganisms, which significantly affect the maintenance of the facial skin epibiome balance.

## Discussion

The contents of various bioactive compounds and their antioxidant activities in tomato pomace extracts and oils have been studied, especially for applications in the food industry^3,6,8,17^. Moreover, it has been shown that these by-products also have broad antibacterial activity against *Escherichia coli, Shigella flexneri, Proteus mirabilis, Staphylococcus aureus*, *Enterococcus faecalis*, and *Acinetobacter baumannii*, which is consistent with the effect we noted of cream with tomato pomace oil on the epibiome species composition^18^.

Despite the unique and valuable properties of tomato pomace extracts and oils, to the best of our knowledge, there are few studies on their use in skincare products. Vasyliev et al.^19^ and Jamaladdine et al.^20^ used tomato pomace extracts to prepare various cosmetic formulations, namely, peel-off masks, water-soluble masks, lip balm, and moisturizing cream. In both cases, the authors focused on the use of deep eutectic solvents but did not examine the effect of the products and tomato pomace extracts on the skin microbiota. Therefore, in the present study, the possibility of using tomato pomace oil waste as an ingredient of microbiome-balance cream for daily facial care was demonstrated. Research on the skin microbiome is often omitted because the microenvironment of the facial microbiome is an open system, which leads to large differences in the bacterial microflora between individuals.

The microbiome of the volunteers was dominated by bacterial classes typical of facial skin: *Acidobacteriota*, *Actinobacteriota,* and *Firmicutes*^21^. Some studies also indicate a significant percentage of bacteria from the *Bacteroidot*a phylum^22^, the percentage of which did not exceed 3% in the case of the analysed volunteers (both male and female). Typical species of bacteria were detected in swabs from the face, mainly *Lactobacillus, Vellionella, Staphylococcus, Cutibacterium, Corynebacterium, Kocuria*, and *Micrococcus*. Most of these aforementioned bacteria constitute the natural, autochthonous microbiota of facial skin. According to Sfirso et al.^23^, the skin microflora is characterized by a large variety of Gram-positive bacteria, such as *Staphylococcus, Cutibacterium,* and *Corynebacterium,* while Gram-negative bacteria are rather considered transitional microorganisms.

*Cutibacterium* spp., which dominated in the tested facial skin samples as lipophilic resident, can utilize sebum; therefore, it preferentially colonizes sebaceous areas, especially pilosebaceous units^24^. In turn, *Corynebacterium* spp. and *Staphylococcus* spp. are found in moist regions of the skin. *Staphylococcus* spp. tolerate the acidic pH found in sebaceous skin. Moreover, they produce lipases, which allows them to utilize the lipid-rich substrate of the skin^25^. Therefore, it can be assumed that the balance of skin microbiome is regulated at the microenvironmental level by physiological and abiotic features (among others, also application of skin care creams). Furthermore, the stability and function of the skin microbial community are driven by host epithelial and immune cells and microbial interactions^26^. A minor fraction of the facial skin microbiota consisted of *Haemophilus*, *Acinetobacter, Enhydrobacter, Moraxella,* and *Pseudomonas*. Similar results were obtained by Myles et al.^27^, who investigated the presence of culturable, gram-negative bacteria from human skin by employing culture-based methods.

The facial skin microflora helps maintain homeostasis and prevents external bacterial infections. For each person, the skin microbial balance is dynamic, changing across the lifespan and dependent on the environment^26^. Unfavourable changes in microflora are strongly related to, among other factors, air pollution^28^. Therefore, the application of a microbiome-friendly cream can be crucial for restoring the structure and function of the skin microbiome after repeated external damage and exposure to air pollutants. The application of cream with tomato pomace oil caused the growth of the dominant genera *Staphylococcus*, *Anaerococcus*, and *Cutibacterium* in both volunteers. However, the abundance of *Cutibacterium* spp. increased in the woman's microbiota, while in the man, it remained at a constant level. Both *Cutibacterium acnes* and *Staphylococcus epidermidis* have been proven to stimulate human keratinocytes and sebocytes to produce antimicrobial peptides that have a positive effect on skin health. In addition, they synthesize bacteriocins that inhibit the growth of pathogens, and the presence of these bacterial species is associated with maintaining the balance of the skin microbiome^29^.

Furthermore, the application of cream containing tomato pomace oil limits the development of potentially opportunistic pathogens, namely, *Kocuria* spp., *Micrococcus* spp., *Veillonella* spp., and *Rothia* spp.^30–33^. Moreover, bacteria from the genera *Micrococcus* and *Kocuria* are widely distributed in the environment, including in soil, air, and water^30,31^. The reduction in their share in the volunteers' epibiome after a 7-day application of the cream enriched with tomato pomace oil indicates the protective effect of this cosmetic by limiting the colonization of the facial skin by environmental microorganisms.

It seems that the beneficial effect of the face cream with tomato pomace oil may be due to the high content of linoleic acid in the tested oil, for which positive effects when applied to the skin were previously reported. Topical application of linoleic acid effectively lightens ultraviolet-induced hyperpigmentation of the skin by suppressing melanin production and enhancing the desquamation of melanin pigment from the epidermis^34^, and significantly reduces the size of acne microcomedones, potentially acting as a comedolytic agent in acne-prone patients^35^. Other studies have shown that topical linoleic acid-rich phosphatidylcholine helps normalize follicular hyperkeratinisation and has an anti-inflammatory effect on acne^36^. Other beneficial effects of this acid on the skin include skin abnormalities correction and a reduction in epidermal DNA synthesis to normal values^37^, treatment and prevention of psoriasis^38^, a decrease in the melasma area^39^, and a skin whitening effect on hyperpigmented skin^40^.

The beneficial effects of active ingredients found in tomato on skin biophysical parameters are consistent with the results obtained in this study, particularly in terms of improving skin inflammation, sensitivity, and tonality. As shown in a double-blind clinical study on a group of 60 women, oral intake of tomato derived carotenoids statistically significantly improved skin barrier strength, firmness and elasticity^41^. Supplementation with lycopene-rich tomato extract has also been reported to have positive effect on facial skin parameters, demonstrating a significant improvement in skin tonality, lines and wrinkles, pore size, smoothness, and skin firmness^42^. Supplements are considered to have an advantage over cosmetics with the same active substances since topical nutrients have limited capacity to penetrate the outer skin layers and have only a local effect. However, a recent in vivo study demonstrated that lycopene emulgel significantly enhanced skin hydration and elasticity, while erythema, melanin, and sebum levels were reduced^43^. Similarly, in this study, topically applied facial cream with tomato pomace oil influenced anti-pollution protection and some biophysical parameters of facial skin, as well as the structure of the skin microbiome.

According to the literature, linoleic acid usually constitutes more than half of the total amount of fatty acids in tomato pomace oil, regardless of the extraction method and differences in the origin of the oil samples^44–46^. Another characteristic feature of tomato pomace oil is that the content of monounsaturated acids is determined by the amount of oleic acid, and the content of saturated acids is determined by the amount of palmitic and stearic acid. Such a correlation also occurred in the oil tested in this study, although the contents of both oleic acid and palmitic and stearic acids were lower than the values presented by other authors by 3.6–7.7%, 2.4–11.9%, and 1.9–4.8%, respectively. However, the tomato pomace oil we tested contained as much as 63.6% linoleic acid, which is higher than that reported in the literature^44–46^.

To our knowledge, the composition of aromatic compounds in tomato pomace oil has not yet been studied. The published data mainly concern volatiles isolated from tomato juice, fresh tomato fruits, tomato pomace extract and cold-pressed seed oil. Differences in the quantitative and qualitative compositions of volatile compounds were detected among the investigated materials. These differences could be caused not only by the nature of the research material but also by the choice of volatile constituent analysis method. Nevertheless, some similarities can be observed. According to published data, nonterpenoid aldehydes (e.g., benzaldehyde, hexanal, butanal, decanal, and acetaldehyde), ketones (e.g., octan-3-one), alcohols (e.g., ethanol and isoamyl alcohol) and acids (e.g., acetic acid, hexanoic acid and heptanoic acid) are the most abundant groups of volatiles in tomato products^47–50^. However, according to Goodman et al.^47^, only hexanol, (Z)-hex-3-enol, and (E)-ex-3-enol are responsible for the characteristic flavour of tomatoes. In the tomato pomace oil tested in this study, terpenoids, phenylpropanoids and nonterpene compounds were found, with the highest content of compounds from the last group (41.6%).

Terpenoids are considered the most important group of scent volatiles that are responsible for the biological activity of essential oils^51^. In the tested tomato pomace oil, carvotanacetone (25.8%) was the major volatile compound identified. This compound is very well known for its antioxidant and antibacterial activity^52^. Interestingly, according to our knowledge, carvotanacetone has not yet been identified in any tomato product, including tomato pomace oil^47–50^. Phenylpropanoids (1.4%) are a very important group of volatiles in tomato pomace oil. These constituents often appear in many essential oils (e.g., clove oil) and show antioxidant and antibacterial activity^53^. To our knowledge, this group has not yet been identified in any tomato product^47–50^.

The presence of compounds with antioxidant activity in the tomato pomace oil may explain the antipollution effect of the face cream with this oil observed in some of the volunteers in this study. Antipollution skin care is an important new trend in cosmetics and is currently gaining popularity around the world. New products, in which substances of botanical origin, especially algae and seed plants, are most often used as antipollution ingredients, are constantly being developed^14^. The antipollution effect of these cosmetics is believed to result from their antioxidant activity. To the best of our knowledge, there are no published data on the use of tomato by-products in this area.

In conclusion, tomato pomace oil can be used as a protective ingredient for face creams with a protective effect, improving the condition of the facial skin, especially by reducing inflammation and skin sensitivity and unifying the skin tone. In addition, face cream with tomato pomace oil can have a beneficial effect on the microbiome, maintaining the so-called microbiome balance, especially in the case of air pollution. This property is particularly desirable since the skin microbiome has been proven to be an integral part of the skin's protective layer.

In this context, further tests should focus on the use of 3D molecular topographical maps combined with omics technologies (metagenomics and metabolomics). These analyses will allow for a better understanding and monitoring of the interactions among the chemical profile of the skin, the microbiome and the metabolites produced by it during regular use of the cream with tomato pomace oil. This will allow the design of a more personalized product to maintain skin epibiome homeostasis.

## Methodology

### Preparation of tomato pomace oil and face creams

#### Oil extraction

Seventy grams of dried tomato pomace was crushed using an electric grinder (Blaupunkt FCG701, Hildesheim, Germany) and then placed in thimbles, which were placed in a Soxhlet apparatus. Four hours of extraction was carried out using hexane (Chempur, Piekary Śląskie, Poland) as a solvent. The obtained extracts were concentrated using a rotary evaporator (IKA Werke HB4 Basic, Staufen im Breisgau, Germany) at 40 mmHg and then mixed. The samples were stored at 4°C for further analyses.

#### Cosmetic emulsion preparation

Both the basic cream and the cream enriched with tomato pomace oil were prepared in 300 g quantities, as detailed in Table 3. The components comprising phase A and phase B were individually weighed, transferred into beakers, and heated to 80 °C, ensuring complete dissolution of the components. Subsequently, the phases were combined by adding phase B to phase A. Homogenization was performed using a POLYTRON® PT 2500 E homogenizer (Kinematica AG, Malters, Switzerland) at 2000 rpm for 2 min. The resulting emulsion was further mixed at 200 rpm until cooled to 40 °C utilizing a WiseStir HS-100D mixer (Witeg Labortechnik GmbH, Wertheim, Germany). In the case of the base cream, fragrance and preservatives were added after reaching the specified temperature. However, no fragrance was incorporated into the cream enriched with tomato pomace oil, as the oil imparted a pleasant scent to the product. Following cooling to room temperature, the pH of the samples was adjusted to 5.5.

**Table 3:** Ingredients and their content in the face creams.

Phase	Ingredient	Content in the basic cream (%)	Content in the cream with tomato pomace oil (%)
A	Cetearyl olivate, Sorbitan olivate	8.00	8.00
	Canola oil	13.00	10.00
	Tomato pomace oil	0	3.00
	Isopropyl myristate	5.00	5.00
	Bees wax	2.00	2.00
B	Water	70.50	71.00
C	Fragrance	0.50	0.00
	Dehydroacetic acid, benzyl alcohol, water	1.00	1.00

### Sample preparation and GC‒MS analysis

#### Fatty acid determination

Fatty acids, assayed as fatty acid methyl esters (FAMEs), were determined in triplicate, and the standard deviation was calculated. Oil samples weighing 100 mg were introduced into round-bottom flasks with a volumetric capacity of 50 mL. Subsequently, 4 mL of a 0.2 M sodium hydroxide solution in methanol and 0.5 mL of a nonadecanoic acid standard were added. The flasks were subjected to reflux conditions for 30 min at the boiling point of the reaction mixture. Next, 5 mL of heptane was injected into the heated samples. Upon cooling, 20 mL of saturated sodium chloride solution was incorporated, facilitating phase separation. Subsequently, 2 mL of the organic phase was isolated and desiccated using anhydrous magnesium sulphate. The samples were subjected to gas chromatography‒mass spectrometry-flame ionization detection (GC‒MS-FID) analysis. The fatty acid methyl ester profiles were determined via gas chromatography equipped with a flame ionization detector (FID) and a Trace GC Ultra mass spectrometer (Thermo Scientific, Waltham, Massachusetts, USA). For analysis, a Stabilwax DA capillary column (Restek Corporation, Bellefonte, USA) with dimensions of 30 m length, 0.25 mm inner diameter, and a stationary phase film thickness of 0.25 μm was used. The temperature program involved ramping from 110 °C (maintained isothermally for 2 min) to 240 °C (held isothermally for 30 min) at a rate of 12 °C/minute. Helium served as the carrier gas at a constant flow rate of 1 ml/min. The ion source temperature was maintained at 200°C throughout the analysis.

#### Volatile compounds isolation and analysis

For analysis of volatile compounds from tomato pomace oil, the HS-SPME-GC-FID-MS technique was used. Sampling was carried out using SPME grey fibre 50/30 μm DVB/CAR/PDMS, stable flex 2 cm (Supelco, Bellefonte, PA, USA) and red fibre 100 µm polydimethylsiloxane coating (Supelco, Bellefonte, PA, USA). One millilitre of tomato pomace oil was introduced to a 15 mL amber vial with a PTFE/Silicone Septa hole cap (Supelco, Bellefonte, PA, USA). Directly before extraction, every sample was incubated at 60 °C for 30 min. Then, the fibre was introduced into the vial, and a 30 min extraction was performed. During the incubation and extraction process, the oil was mixed with a 3 mm magnetic stirrer at a speed of 300 rpm. After extraction, the fibre was directly transferred to the injector of the GC-FID-MS apparatus (Thermo Fischer Scientific Inc., Waltham, Massachusetts, USA). The desorption of volatile compounds lasted 10 min. Analysis was performed in two repetitions, and the average percentages were taken. For the investigation, a Trace GC Ultra gas chromatograph coupled with a DSQ II mass spectrometer (Thermo Fischer Scientific Inc., Waltham, Massachusetts, USA) was used. Simultaneous GC‒FID‒MS analysis was performed using an MS‒FID splitter (SGE, Analytical Science, Austin, TX, USA). A nonpolar capillary column, Rtx-1 ms (60 m × 0.25 mm, 0.25 μm film thickness), was used for volatile analysis. The temperature program was as follows: 50 °C (3 min), from 4 °C/min to 300 °C; injector (SSL) temperature, 280 °C; detector (FID) temperature, 300 °C; and transfer line temperature, 250 °C. Helium was used as the carrier gas with a constant flow pressure of 200 kPa and a split ratio of 1:20. The mass spectrometer program was as follows: ion source temperature, 200 °C; ionization energy, 70 eV (EI); scan mode, full scan; and mass range, 33–420. The percentages of constituents were computed from the GC peak area without using correction factors. Identification of the components was based on a comparison of their mass spectra and linear retention indices (RI, nonpolar column), determined with reference to a series of n-alkanes C8-C24 with literature data, computer libraries NIST 2011, MassFinder 4.1 and Adams^50^.

### Tests of face cream with participants

#### Participant recruitment and study design

A heterogeneous group of volunteers was recruited for the study, i.e., 3 men and 7 women aged 25–70 years. Ethical approval was waived in accordance with Rules and Regulations of the Research Ethics Committee of Lodz University of Technology. All participants provided written informed consent to participate in the study. Participants had to meet the following conditions: not have any systemic or skin disorders, such as acne, psoriasis, atopic dermatitis or infectious skin diseases; and not use topical or systemic antibiotics within the last month. Participants were asked to use a basic cream on the left side of the face and a cream with tomato pomace oil on the right side twice a day (morning and evening) for 7 days. During this time, participants were not allowed to use any other skin care cosmetics or take antimicrobial preparations or medications that could interfere with the study results. They were allowed to maintain their own skincare routines, such as skin cleansing, makeup or shaving.

#### Antipollution properties of the face cream

The antipollution effect of tomato pomace oil was determined by assessing whether regular application of the tested face cream for 7 days reduced the attraction and penetration of particulate pollutants on the skin surface and within the skin pores. For this purpose, the sebum pollution model proposed by Peterson et al.^16^ was used (Table 4).

**Table 4:** Composition of the sebum pollution model (SPM).

INCI	Content (%)
Polyglyceryl oleate	2.5
Cholesterol	3.5
Squalane	7.0
Oleic acid	15.0
Glyceryl trioleate	15.0
Cetyl palmitate	8.0
Mineral oil	10.0
Petrolatum	10.0
Water	20.0
Carbon black (CI 77,266)	4.0
Iron oxides (CI 77,492)	4.0
Preservative	1.0

Circle-shaped areas with a diameter of 5 cm were drawn on the apples of both cheeks of the participants. Equal amounts of the sebum pollution model (0.8 g) were applied evenly with sterile spatulas on the circle marked on each cheek. After 10 min, the mixture was washed off using cotton pads, each of which was soaked in 2 ml of a 25% solution of commercially available face wash gel (Soraya Plante, Bielenda Kosmetyki Naturalne, Poland). To remove sebum pollution model one cotton pad was used for each cheek.

The evaluation test was non-invasive with the use of pre- and post-cleaning photographic documentation and visual assessment and was based on a three-point scale: 1—the skin surface remained slightly grey with single visible contaminated pores, 2—the skin surface remained slightly grey with more intense local residues of pollutants and visible contaminated pores, and 3—the skin surface remained visibly grey with very intense local residual pollutants and visible contaminated pores (Fig. 1).

#### Skin biophysical parameters

On the eve of the study, the volunteers applied standard evening facial care, and from then on, participants were not allowed to perform any facial skin care activities until the analysis was carried out the next morning. Skin biophysical parameter measurements were performed independently for each half of the face using an Aram Huvis v 0.4.9 skin analyser and Wizard computer software (Aram Huvis Co., Jungwon-gu, Rep. Korea). Skin parameters, including moisture, elasticity, pore, melanin, inflammation, and sensitivity, were measured twice—at the beginning of the study (D0) and after 7 days (D7) of the tested face creams application.

#### Skin microbiome sample collection

Samples for skin microbiome analysis were collected on the first day of the experiment (D0) and after 7 days (D7) of regular use of basic cream and cream with tomato pomace oil by the participants. Samples were collected via the swabbing method with a cotton swab soaked in 0.9% sodium chloride with 0.1% Tween-20 in a Z-stroke manner^55^. Until the swabs were taken, the participants were not allowed to perform any facial skin care activities since the previous evening. The swab at D0 was taken from the entire face surface, and at time D7 from each half of the face independently.

### Skin microbiome analysis

#### DNA extraction

Total genomic DNA was extracted from the facial swabs of the probands using the Swab Genomic DNA Isolation Kit (A&A Biotechnology, Gdańsk, Poland). The tips of the swabs containing the collected material was cut off and placed in tubes. Then, lysing solution was added so that the swabs were completely immersed. Further DNA extraction steps were performed following the manufacturer’s protocol. The quality and quantity of the extracted DNA were assessed using a Qubit Fluorometer (Thermo Fisher Scientific, Waltham, MA, USA), and the genomic DNA was stored at − 20 °C.

#### 16S rDNA library preparation and high-throughput sequencing

The primer sets 341F and 785R^56^ targeting the V1-V2 hypervariable region of the 16S rDNA gene were used. PCR was carried out with Q5 Hot Start High-Fidelity 2 × Master Mix (New England BioLabs Inc., Ipswich, MA, USA) under conditions consisting of initial denaturation for 120 s at 95 °C, followed by denaturation for 30 s at 95 °C, annealing for 30 s at 55 °C, extension for 60 s at 72 °C (25 cycles), and a final extension for 5 min at 72 °C. The PCR products were purified by AMPure XP magnetic beads (Beckman Coulter Life Sciences Inc., Indianapolis, IN, USA), and the amplicon libraries were indexed using a Nextera Index Kit (Thermo Fisher Scientific, Waltham, MA, USA). Sequencing on the Illumina MiSeq platform (San Diego, CA, USA) was performed by Genomed S.A. (Warsaw, Poland) via paired–end technology (PE, 2 × 300 nt).

#### Bioinformatic analysis of the microbial community

Preliminary data analysis was performed on a MiSeq System (Illumina, Inc., San Diego, CA, USA) with MiSeq Reporter (MSR) v 2.6 software (Illumina Inc., San Diego, CA, USA). The QIIME2 2021.4 bioinformatic platform (https://qiime2.org/), which is based on the SILVA v 138 database, was used for the readings classification. Briefly, analysis included the following steps: removal of adapter sequences, evaluation of the quality of the readings and removal of low-quality sequences, connecting paired sequences, clustering based on the selected database of reference sequences, and removal of sequence chimeras^57^. Operational Taxonomic Units (OTUs) were assigned to taxa in the selected database of reference sequences at an identity threshold of 97%.

The diversity of the facial skin microbial community was analysed with alpha diversity estimators, including the Shannon index, and calculated by the Mothur program v 1.30.1. The similarities and differences among the samples are presented on Venn diagrams generated with Venny 2.1 software. Microbial networks were constructed using the Gephi program v 0.10.

### Statistical analysis

The results were expressed as the arithmetic mean ± standard deviation from three independent determinations. In Figs. 1 and 2, the results were presented as box-and-whisker plots based on the five-number summary: the minimum, the maximum, the statistical median, and the first and third quartiles. The significance of differences between means was determined using analysis of variance (one-way ANOVA; OriginPro 8.1, OriginLab Corporation, Northampton, MA, USA) and Tukey HSD test, with p ≤ 0.05.

## Data Availability

The datasets generated and/or analyzed during the current study are available in the NCBI SRA database with accession number PRJNA1126159.
